# Supplementary material for: The Molecular Determination of Hybridity and Homozygosity Estimates in Breeding Populations of Lettuce (Lactuca sativa L.)
Source: Genes (Basel). 2019 Nov 9;10(11):916. doi: 10.3390/genes10110916 (PMC6895879; doi:10.3390/genes10110916)
Supplement: Supplementary file 1 [file genes-10-00916-s001.zip › Supplementary/Supplementary Figures[19100].docx]

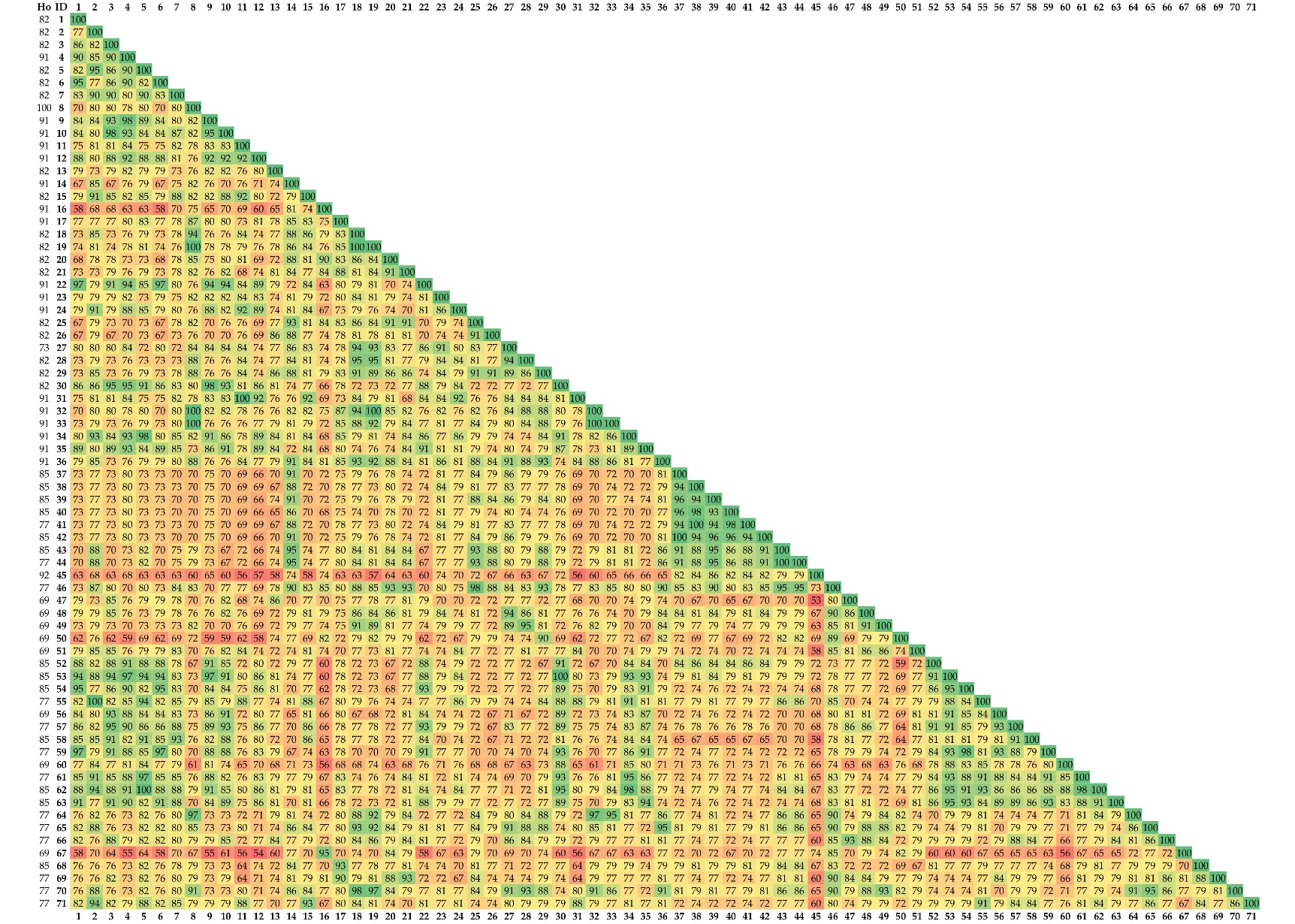


**Figure S1.** Pairwise genetic similarity matrix of the 71 individuals analysed (in percentages) based on Rohlf’s genetic similarity coefficient. High genetic similarity values are labelled in green, the low values in red, and intermediate values are coloured on a scale from green to red. The observed homozygosity values of the 71 putative parental lines are reported to the left of each ID name.


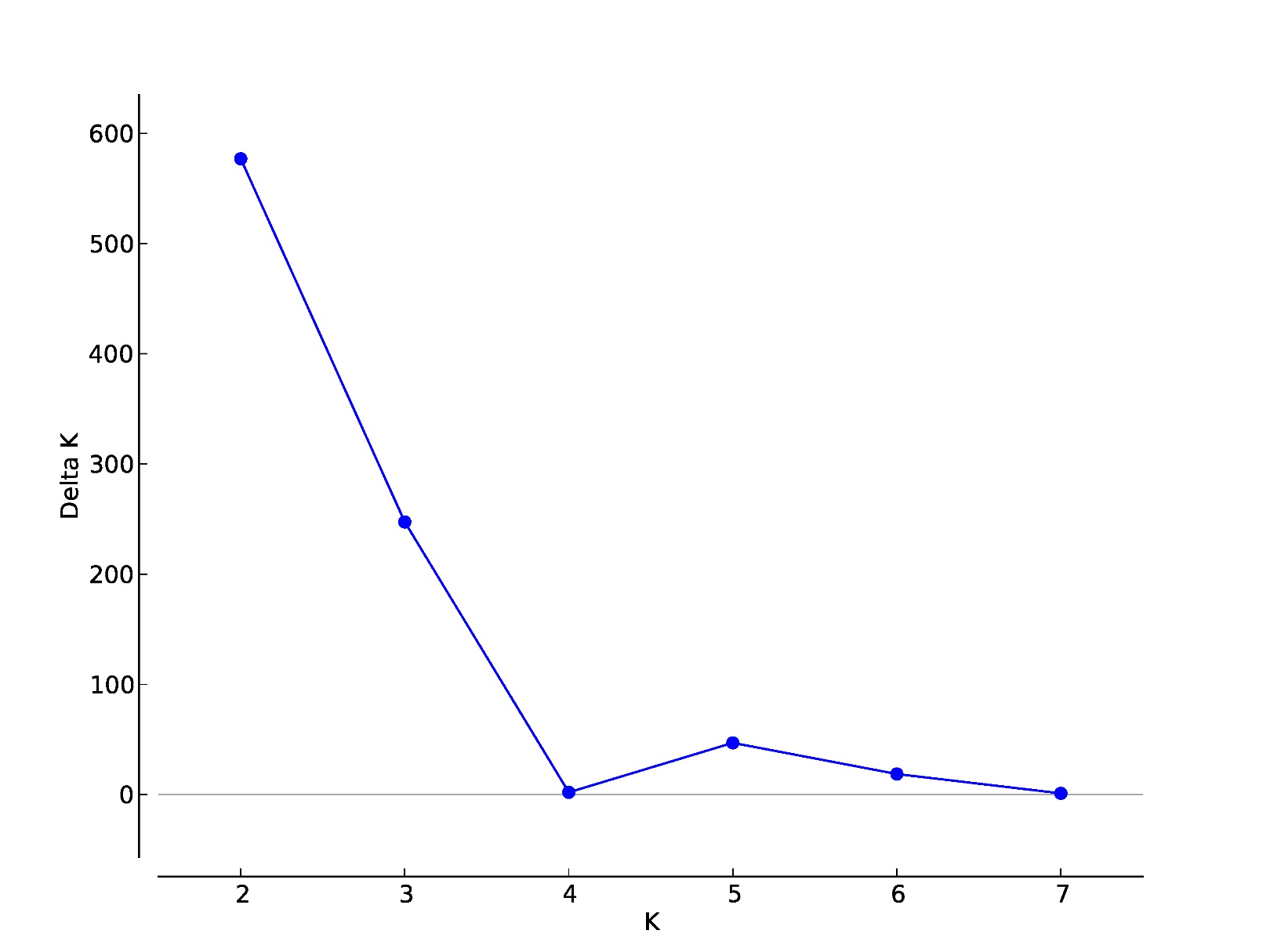


**Figure S2.** Definition of the subgroup number of parental lines based on the SSR marker dataset. Mean ∆K is calculated as |L” (K)|/(SD(L(K)), following Evanno et al*.* [23]. The blue line represents the ∆K values.

**
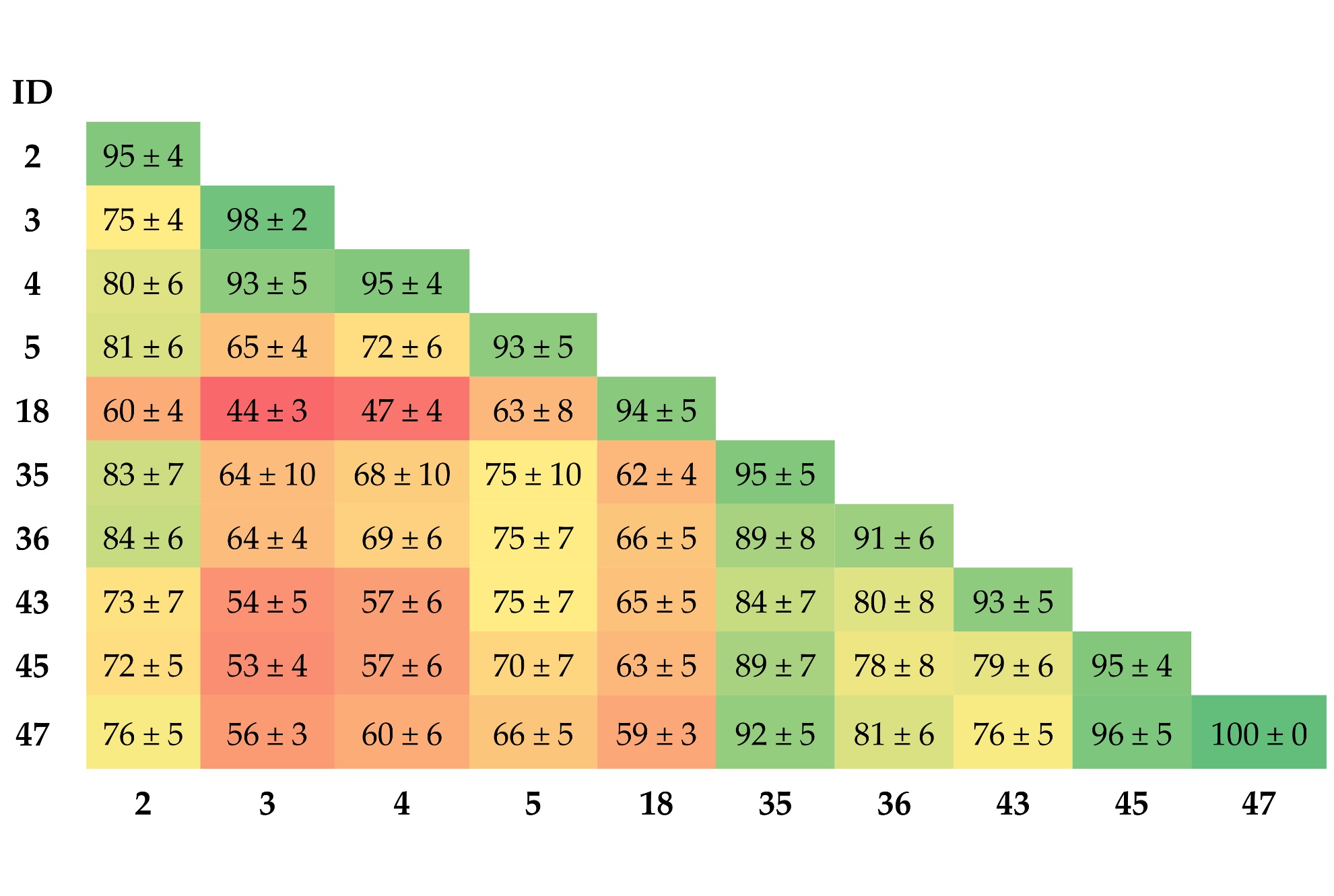
**

**Figure S3.** Pairwise genetic similarity matrix of ten selected populations (in percentages) based on the Jaccard coefficient. The high genetic similarity values are labelled in green, the low values in red, and intermediate values are coloured on a scale from green to red.
