# Supplementary material for: The Molecular Determination of Hybridity and Homozygosity Estimates in Breeding Populations of Lettuce (Lactuca sativa L.)
Source: Genes (Basel). 2019 Nov 9;10(11):916. doi: 10.3390/genes10110916 (PMC6895879; doi:10.3390/genes10110916)
Supplement: Supplementary file 1 [file genes-10-00916-s001.zip › Supplementary/Supplementary Tables.docx]

**Table S1:** Lettuce parental lines information, including ID of accessions, cultivar type of materials and subpopulation classification based on STRUCTURE analysis (1 = blue and 2 = red).

| **Parental ID** | **Cultivar type** | **STRUCTURE subpopulation** |
| --- | --- | --- |
| 1 | butterhead | 1 |
| 2 | butterhead | 1 |
| 3 | butterhead | 1 |
| 4 | butterhead | 1 |
| 5 | butterhead | 1 |
| 6 | butterhead | 1 |
| 7 | butterhead | 1 |
| 8 | batavia | 2 |
| 9 | butterhead | 1 |
| 10 | butterhead | 1 |
| 11 | butterhead | 1 |
| 12 | butterhead | 1 |
| 13 | butterhead | 1 |
| 14 | butterhead | 2 |
| 15 | romaine | 2 |
| 16 | batavia | 1 |
| 17 | romaine | 2 |
| 18 | romaine | 2 |
| 19 | romaine | 2 |
| 20 | romaine | 2 |
| 21 | iceberg | 2 |
| 22 | iceberg | 1 |
| 23 | iceberg | 2 |
| 24 | iceberg | 1 |
| 25 | butterhead | 2 |
| 26 | butterhead | 2 |
| 27 | butterhead | 2 |
| 28 | butterhead | 2 |
| 29 | butterhead | 2 |
| 30 | butterhead | 1 |
| 31 | butterhead | 1 |
| 32 | batavia | 2 |
| 33 | batavia | 2 |
| 34 | butterhead | 1 |
| 35 | butterhead | 1 |
| 36 | leaf | 2 |
| 37 | leaf | 2 |
| 38 | leaf | 2 |
| 39 | leaf | 2 |
| 40 | leaf | 2 |
| 41 | leaf | 2 |
| 42 | leaf | 2 |
| 43 | leaf | 2 |
| 44 | leaf | 2 |
| 45 | leaf | 2 |
| 46 | butterhead | 2 |
| 47 | butterhead | 2 |
| 48 | leaf | 2 |
| 49 | leaf | 2 |
| 50 | leaf | 2 |
| 51 | butterhead | 1 |
| 52 | butterhead | 1 |
| 53 | butterhead | 1 |
| 54 | butterhead | 1 |
| 55 | butterhead | 1 |
| 56 | butterhead | 1 |
| 57 | butterhead | 1 |
| 58 | butterhead | 1 |
| 59 | butterhead | 1 |
| 60 | butterhead | 1 |
| 61 | butterhead | 1 |
| 62 | butterhead | 1 |
| 63 | butterhead | 1 |
| 64 | butterhead | 2 |
| 65 | butterhead | 2 |
| 66 | romaine | 2 |
| 67 | romaine | 2 |
| 68 | romaine | 2 |
| 69 | romaine | 2 |
| 70 | romaine | 2 |
| 71 | romaine | 1 |

**Table S2:** SSR primer tails and dyes. List of the primer tails used with their sequences and corresponding dyes.

| **Universal primer** | **Sequence 5'-3'** | **Dye** |
| --- | --- | --- |
| M13 | TTGTAAAACGACGGCCAGT | 6-FAM |
| PAN1 | GAGGTAGTTATTGTGGAGGAC | VIC |
| PAN2 | GGAATTAACCGCTCACTAAAG | NED |
| PAN3 | TGTAGAAAGACGAAGGGAAGG | PET |

**Table S3:** Lettuce plant material information, including ID of accessions used in the crosses, total number of plants per programmed cross, number of informative marker loci, hybrid plants, selfed plants and unexpected genotypes, and the mean hybridisation values (in percentages) for all the programmed crosses.

| **ID Cross** | **No plants analysed*** | **Informative marker loci** | **No hybrid plants** | **No selfed plants** | **No unexpected genotypes** | **Hybridisation (%)** |
| --- | --- | --- | --- | --- | --- | --- |
| **1 × 6** | 10 | 2 | 0 | 10 | 0 | 0 |
| **1 × 34** | 10 | 2 | 10 | 0 | 0 | 100 |
| **1 × 35** | 8 | 1 | 7 | 1 | 0 | 88 |
| **1 × 5** | 10 | 2 | 0 | 10 | 0 | 0 |
| **6 × 30** | 11 | 1 | 8 | 0 | 3 | 100 |
| **6 × 34** | 12 | 2 | 8 | 0 | 4 | 100 |
| **30 × 34** | 8 | 1 | 6 | 2 | 0 | 75 |
| **7 × 6** | 10 | 4 | 4 | 6 | 0 | 40 |
| **7 × 34** | 10 | 3 | 4 | 6 | 0 | 40 |
| **7 × 35** | 10 | 2 | 10 | 0 | 0 | 100 |
| **7 × 5** | 9 | 2 | 3 | 0 | 6 | 100 |
| **35 × 6** | 9 | 1 | 8 | 1 | 0 | 89 |
| **35 × 30** | 10 | 1 | 3 | 5 | 2 | 38 |
| **35 × 34** | 11 | 2 | 4 | 7 | 0 | 36 |
| **35 × 5** | 9 | 2 | 5 | 4 | 0 | 56 |
| **10 × 6** | 11 | 2 | 7 | 4 | 0 | 64 |
| **10 × 34** | 11 | 2 | 7 | 4 | 0 | 64 |
| **10 × 5** | 9 | 2 | 3 | 6 | 0 | 33 |
| **36 × 8** | 12 | 3 | 8 | 4 | 0 | 67 |
| **11 × 12** | 8 | 2 | 7 | 1 | 0 | 88 |
| **22 × 34** | 10 | 3 | 6 | 4 | 0 | 60 |
| **12 × 31** | 10 | 1 | 5 | 5 | 0 | 50 |
| **2 × 6** | 9 | 3 | 3 | 6 | 0 | 33 |
| **2 × 35** | 10 | 3 | 4 | 6 | 0 | 40 |
| **2 × 5** | 9 | 2 | 9 | 0 | 0 | 100 |
| **3 × 6** | 8 | 3 | 0 | 8 | 0 | 0 |
| **3 × 35** | 10 | 2 | 7 | 3 | 0 | 70 |
| **3 × 5** | 10 | 1 | 1 | 9 | 0 | 10 |
| **33 × 8** | 9 | 1 | 8 | 1 | 0 | 89 |
| **15 × 18** | 11 | 2 | 3 | 3 | 5 | 50 |
| **15 × 19** | 11 | 1 | 3 | 7 | 1 | 30 |
| **16 × 18** | 10 | 4 | 3 | 5 | 2 | 38 |
| **17 × 16** | 10 | 4 | 0 | 10 | 0 | 0 |
| **17 × 18** | 12 | 4 | 5 | 0 | 7 | 100 |
| **17 × 19** | 8 | 2 | 6 | 0 | 2 | 100 |
| **20 × 18** | 11 | 2 | 6 | 5 | 0 | 55 |
| **20 × 19** | 8 | 2 | 8 | 0 | 0 | 100 |
| **21 × 16** | 12 | 4 | 1 | 11 | 0 | 8 |
| **21 × 18** | 9 | 3 | 1 | 5 | 3 | 17 |
| **21 × 19** | 11 | 2 | 5 | 6 | 0 | 45 |
| **4 × 6** | 10 | 2 | 8 | 2 | 0 | 80 |
| **4 × 34** | 11 | 1 | 10 | 1 | 0 | 91 |
| **4 × 35** | 8 | 1 | 8 | 0 | 0 | 100 |
| **4 × 5** | 10 | 1 | 7 | 3 | 0 | 70 |
| **5 × 6** | 12 | 2 | 11 | 1 | 0 | 92 |
| **5 × 30** | 8 | 1 | 6 | 2 | 0 | 75 |
| **26 × 13** | 12 | 4 | 4 | 2 | 6 | 67 |
| **26 × 14** | 11 | 3 | 8 | 0 | 3 | 100 |
| **26 × 25** | 11 | 3 | 11 | 0 | 0 | 100 |
| **24 × 23** | 10 | 3 | 9 | 1 | 0 | 90 |
| **23 × 24** | 10 | 3 | 10 | 0 | 0 | 100 |
| **27 × 28** | 12 | 2 | 11 | 1 | 0 | 92 |
| **29 × 27** | 12 | 2 | 0 | 10 | 2 | 0 |
| **45 × 41** | 10 | 5 | 10 | 0 | 0 | 100 |
| **45 × 39** | 7 | 3 | 2 | 5 | 0 | 29 |
| **45 × 37** | 10 | 3 | 9 | 1 | 0 | 90 |
| **45 × 40** | 10 | 4 | 10 | 0 | 0 | 100 |
| **45 × 42** | 10 | 4 | 10 | 0 | 0 | 100 |
| **45×38** | 10 | 4 | 3 | 7 | 0 | 30 |
| **45×43** | 10 | 3 | 10 | 0 | 0 | 100 |
| **59 × 61** | 10 | 3 | 3 | 7 | 0 | 30 |
| **57 × 58** | 10 | 1 | 7 | 3 | 0 | 70 |
| **57 × 63** | 9 | 2 | 6 | 3 | 0 | 67 |
| **56 × 61** | 10 | 2 | 10 | 0 | 0 | 100 |
| **56 × 62** | 10 | 2 | 9 | 1 | 0 | 90 |
| **54 × 62** | 9 | 2 | 8 | 1 | 0 | 89 |
| **54 × 60** | 10 | 2 | 10 | 0 | 0 | 100 |
| **54 × 57** | 10 | 4 | 10 | 0 | 0 | 100 |
| **54 × 53** | 9 | 2 | 8 | 1 | 0 | 89 |
| **54 × 55** | 10 | 3 | 0 | 10 | 0 | 0 |
| **54 × 61** | 10 | 2 | 8 | 2 | 0 | 80 |
| **54 × 58** | 10 | 3 | 8 | 2 | 0 | 80 |
| **54 × 56** | 10 | 4 | 8 | 2 | 0 | 80 |
| **54 × 63** | 10 | 2 | 10 | 0 | 0 | 100 |
| **71 × 67** | 10 | 5 | 4 | 6 | 0 | 40 |
| **67 × 66** | 10 | 4 | 10 | 0 | 0 | 100 |
| **68 × 67** | 10 | 5 | 10 | 0 | 0 | 100 |
| **70 × 66** | 10 | 3 | 10 | 0 | 0 | 100 |
| **69 × 67** | 10 | 3 | 3 | 7 | 0 | 30 |
| **51 × 50** | 9 | 5 | 4 | 5 | 0 | 44 |
| **51 × 49** | 6 | 4 | 2 | 4 | 0 | 33 |
| **50 × 48** | 10 | 3 | 9 | 1 | 0 | 90 |
| **50 × 49** | 8 | 5 | 8 | 0 | 0 | 100 |
| **49 × 48** | 8 | 4 | 7 | 1 | 0 | 88 |
| **52 × 50** | 8 | 7 | 8 | 0 | 0 | 100 |
| **52 × 49** | 8 | 7 | 8 | 0 | 0 | 100 |
| **47 × 46** | 8 | 3 | 5 | 3 | 0 | 63 |
| **47 × 44** | 9 | 6 | 8 | 1 | 0 | 89 |
| **65 × 64** | 10 | 2 | 2 | 8 | 0 | 20 |
| **Total** | 871 | 242 | 556 | 269 | 46 |  |
| **Mean** |  |  |  |  |  | 68 |

* **No plants analysed** = (No F1 + No U.G. + No S.P)

**Table S4.** Information about ten F3 selected populations, including ID of parental lines used in the crosses.

| **ID cross** | **ID F3 population** |
| --- | --- |
| 7 x 6 | 2 |
| 7 x 6 | 3 |
| 7 x 6 | 4 |
| 54 x 58 | 5 |
| 7 x 5 | 18 |
| 2 x 6 | 35 |
| 2 x 6 | 36 |
| 2 x 6 | 43 |
| 2 x 6 | 45 |
| 2 x 6 | 47 |
