# Supplementary figures and images for: The Molecular Determination of Hybridity and Homozygosity Estimates in Breeding Populations of Lettuce (Lactuca sativa L.)
Source: Genes (Basel). 2019 Nov 9;10(11):916. doi: 10.3390/genes10110916 (PMC6895879; doi:10.3390/genes10110916)

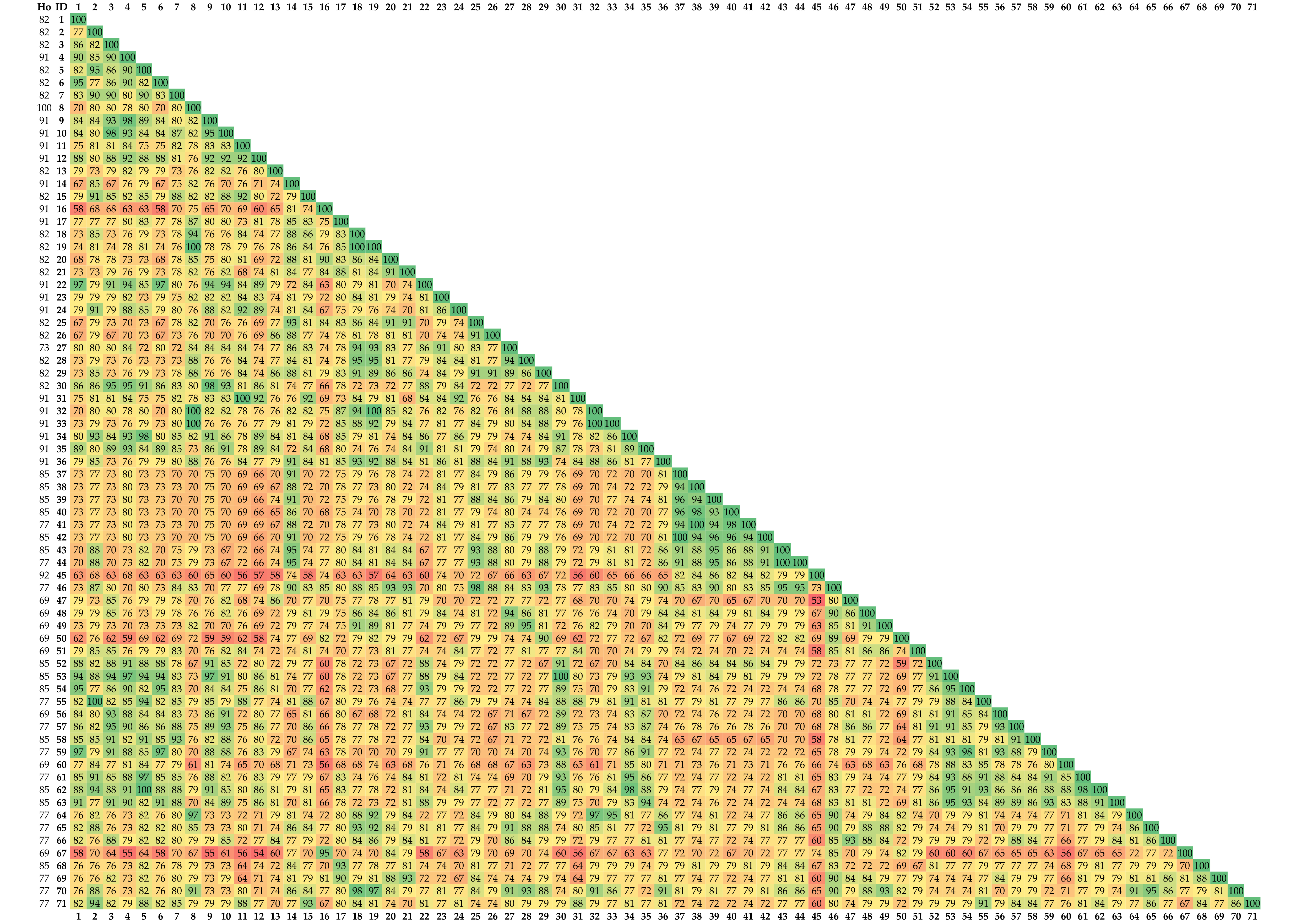

Supplement: Supplementary file 1 [file genes-10-00916-s001.zip › Supplementary/Figure-S1.jpeg]

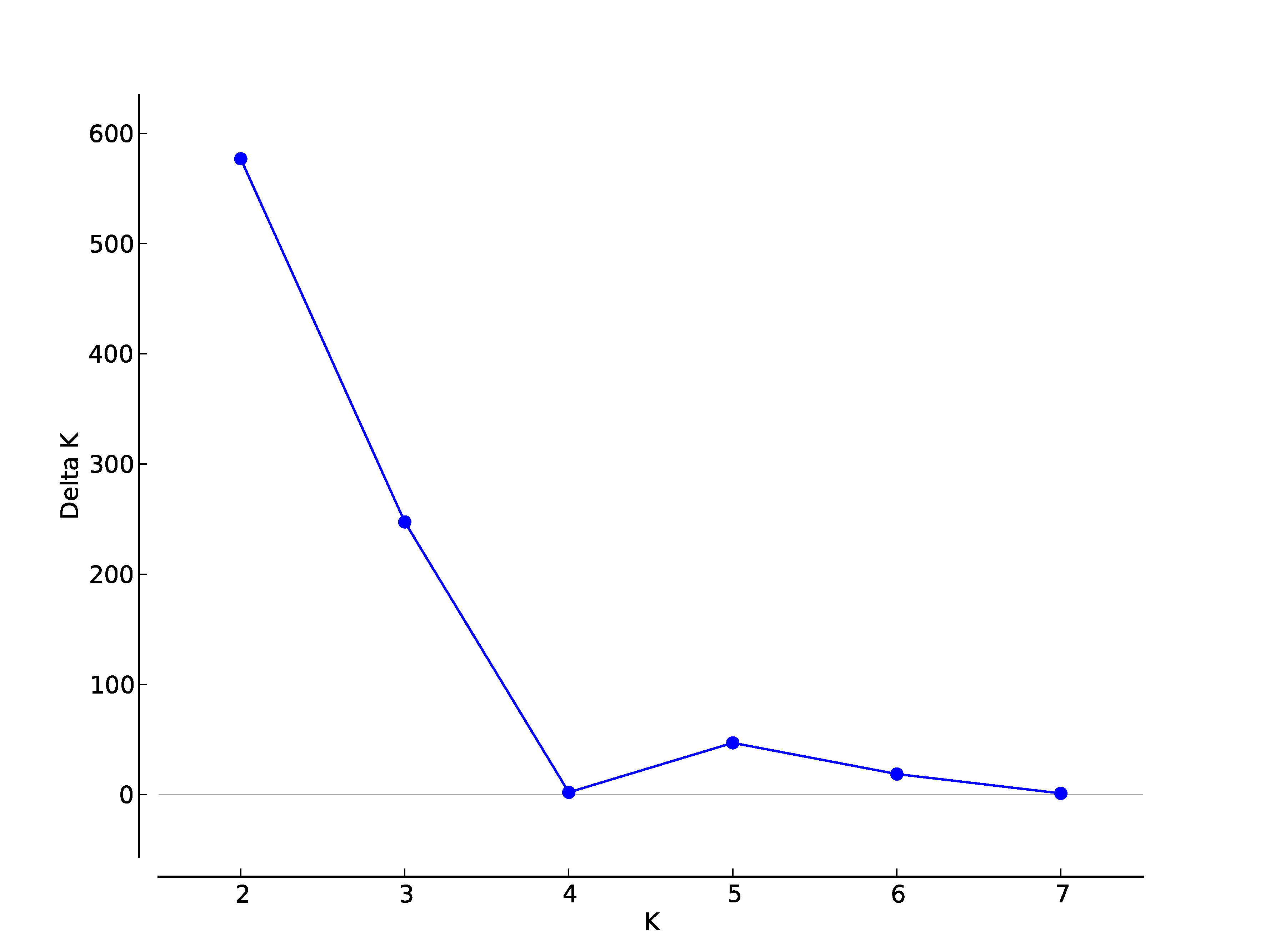

Supplement: Supplementary file 1 [file genes-10-00916-s001.zip › Supplementary/Figure-S2.jpeg]

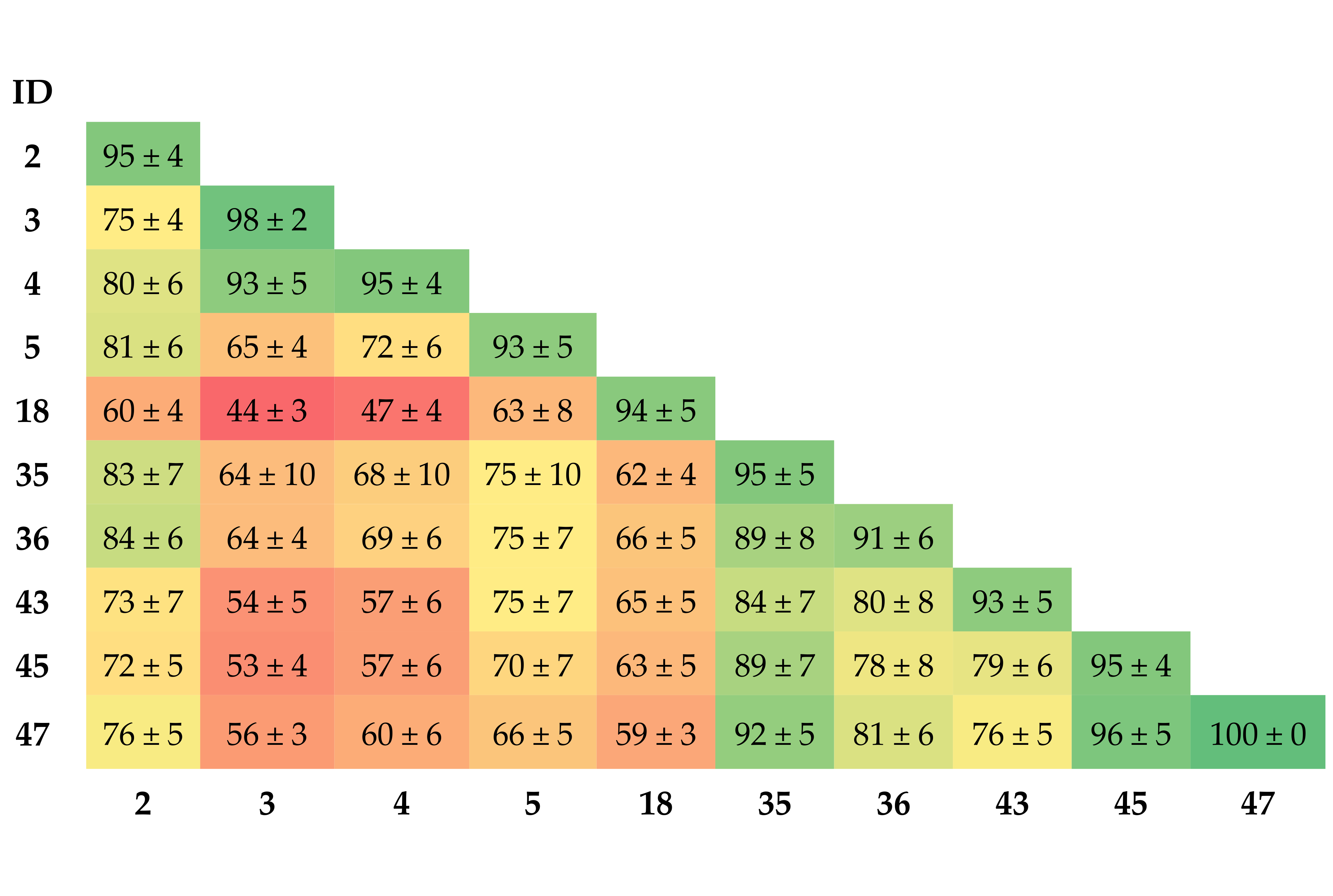

Supplement: Supplementary file 1 [file genes-10-00916-s001.zip › Supplementary/Figure-S3.jpeg]
